# Molecular Organization and Chromosomal Localization Analysis of *5S* rDNA Clusters in Autotetraploids Derived From *Carassius auratus* Red Var. (♀) × *Megalobrama amblycephala* (♂)

**DOI:** 10.3389/fgene.2019.00437

**Published:** 2019-05-15

**Authors:** QinBo Qin, QiWen Liu, ChongQing Wang, Liu Cao, YuWei Zhou, Huan Qin, Chun Zhao, ShaoJun Liu

**Affiliations:** State Key Laboratory of Developmental Biology of Freshwater Fish, College of Life Sciences, Hunan Normal University, Changsha, China

**Keywords:** autotetraploid line, distant hybridization, *5S* rDNA, FISH, chromosomal loci

## Abstract

The autotetraploid fish (4*n* = 200, RRRR) (abbreviated as 4*n*RR) resulted from the whole genome duplication of red crucian carp (*Carassius auratus* red var., 2*n* = 100, RR) (abbreviated as RCC). During investigation of the influence of polyploidization on organization and evolution of the multigene family of *5S* rDNA, molecular organization and chromosomal localization of the *5S* rDNA were characterized in autotetraploid fish. By sequence analysis of the coding region (*5S*) and adjacent non-transcribed spacer (NTS), three distinct *5S* rDNA units (type I: 203 bp; type II: 340 bp; and type III: 477bp) were identified and characterized in 4*n*RR. These *5S* rDNA units were inherited from their female parent (RCC), in which obvious base variations in NTS and array recombination of repeat units were found. Using fluorescence *in situ* hybridization employing different *5S* rDNA units as probes, these *5S* rDNA clusters were localized in chromosomes of 4*n*RR, respectively, and showed obvious loss of chromosomal loci (type I and type II). Our data revealed genetic variation of the *5S* rDNA multigene family in the genome of autopolyploid fish. Furthermore, results provided new insights into the evolutionary patterns of this vertebrate multigene family.

## Introduction

Polyploidy is a significant mode of speciation in eukaryotes (Mallet, 2007; Otto, 2007), especially in vertebrates. Ohno (1970) proposed the genome duplication hypothesis, in which two rounds of whole genome duplication occurred during early vertebrate evolution. Polyploids are generally divided in to categories depending on their chromosomal composition and their manner of formation. The autopolyploids have chromosome sets coming from the genome of one species (e.g., AAAA) and exhibit multivalent pairing during meiosis, while the allopolyploids result from the combination of sets of chromosomes from two or more different taxa (e.g., AABB) and predominantly form bivalent pairings (Comai, 2005). Notably, multivalent pairing may cause meiotic irregularities and result in reduced fertility compared with diploid progenitors (Jackson, 1982; Parisod et al., 2010). Thus, vertebrate autopolyploids are relatively rare compared with allopolyploids, and the influence of autopolyploidization on intragenomic variation is poorly understood.

*5S* ribosomal RNA (rRNA) is a component of the large ribosomal subunit in all ribosomes. In vertebrates, the *5S* ribosomal DNA (*5S* rDNA) is organized in tandem arrays with repeat units composed of a 120-bp coding sequence (*5S*) that encodes the *5S* rRNA and a highly variable non-transcribed spacer (NTS) (Korn and Brown, 1978; Nielsen et al., 1993; Hallenberg and Frederiksen, 2001; Pasolini et al., 2006). Molecular organization and chromosomal localization of the *5S* rDNA have been extensively characterized in bony fish (Iue et al., 1989; Rocco et al., 2005; Qin et al., 2010; Danillo et al., 2011). Polyploidization plays an important role in the evolution of fish. However, the features of the *5S* rDNA have been rarely reported in polyploid fish. Previously, we successfully obtained fertile allotetraploid hybrids (4*n* = 148, RRBB) (abbreviated as 4*n*RB) from the first generation of *Carassius auratus* red var. (2*n* = 100, RR) (♀) × *Megalobrama amblycephala* (2*n* = 48, BB) (♂) hybrids (Qin et al., 2014a). The abnormal chromosomal behavior of allotetraploid hybrids during meiosis leads to the formation of autodiploid sperm and autodiploid ova, and the fertilization of these ova by these sperm in turn produces autotetraploid fish (4*n*RR) (Qin et al., 2014b, 2015a). Autotetraploids produce diploid ova and diploid sperm and maintain the formation of the autotetraploid line (F_1_–F_10_), which could be used as a new model system for investigating the influence of polyploidy on the organization and evolution of the multigene family of *5S* rDNA. In this paper, molecular organization and chromosomal localization of the *5S* rDNA have been characterized in the autotetraploid and their parents (RCC). Obvious loss of chromosomal loci, base variations in NTS, and array recombination of repeat units have been found in the newly established autotetraploidy genomes. Our results extend the knowledge of the influence of polyploidy on the organization and evolution of *5S* rDNA of fish, and are also useful in clarifying aspects of vertebrate genome evolution.

## Materials and Methods

### Source of Samples

All fish were cultured in ponds and fed with artificial feed at the Protection Station of Polyploidy Fish, Hunan Normal University. Fish treatments were carried out according to the regulations for protected wildlife and the Administration of Affairs Concerning Animal Experimentation, and approved by the Science and Technology Bureau of China. Approval from the Department of Wildlife Administration was not required for the experiments conducted in this paper. The fish were deeply anesthetized with 100 mg/L MS-222 (Sigma-Aldrich, St. Louis, MO, United States) before dissection.

### Animals and Crosses

During the reproductive season (April to June) in 2012, the first generation (4*n*RB) of *C. auratus* red var. (♀) × *M. amblycephala* (♂) was produced. During the reproductive season (April to June) of 2014, the second generation (4*n*RR) was produced by self-crossing of 4*n*RB.

### Preparation of Chromosome Spreads

Chromosome counts were performed using kidney tissue from 10 RCC and 10 4*n*RR. After culture for 1–3 days at a water temperature of 18–22°C, the samples were injected with concanavalin one to three times at a dose of 2–8 mg/g body weight. The interval between injections was 12–24 h. Six hours prior to dissection each sample was injected with colchicine at a dose of 2–4 mg/g body weight. The excised kidney tissue was ground in 0.9% NaCl, followed by hypotonic treatment with 0.075 M KCl at 37°C for 40–60 min and then fixed in 3:1 methanol–acetic acid with three changes. The cells were dropped onto cold, wet slides and stained for 30 min in 4% Giemsa. The shape and number of chromosomes were analyzed under a microscope. For each type of fish, 200 metaphase spreads (20 metaphase spreads from each sample) of chromosomes were analyzed. The preparations were examined under an oil lens at a magnification of 3330×.

### PCR Amplification and Sequencing of *5S* rDNA Sequences

Total genomic DNA was isolated from peripheral blood cells according to the standard phenol: chloroform extraction procedure described by Sambrook et al. (1989). To acquire preliminary information on the organization of the *5S* rDNA repeat variants, and to test for the possible coexistence of different repeat units in the same array, DNA samples of 3 RCC and 3 4*n*RR were amplified with primers *5S*P1-*5S*P2R (5′-GCTATGCCCGATCTCGTCTGA-3′ and 5′-CAGGTTGGTAT GGCCGTAAGC-3′) and with primers *5S*NT1-*5S*NT2R (5′-GGCGAGTAGATTGGCTGAACA-3′ and 5′-CAATCTAATCGCCAGTACATTATAT-3′). The PCR reaction was performed in a volume of 25 μL with approximately 20 ng of genomic DNA, 1.5 mM of MgCl_2_, 200 μM of each dNTP, 0.4 μM of each primer, and 1.25 U of Taq polymerase (Takara). The temperature profile was as follows: an initial denaturation step at 94°C for 5 min, followed by 30 cycles of 94°C for 30 s, 56°C for 30 s, and 72°C for 1 min, with a final extension step at 72°C for 10 min. Amplification products were separated on a 3.0% agarose gel using TBE buffer. The DNA fragments were purified using a gel extraction kit (Sangon) and ligated into pMD18-T (Takara). Plasmids were transformed into *Escherichia coli* DH5a, propagated, and then purified. The cloned DNA fragments were sequenced using an automated DNA sequencer (ABI PRISM 3730). Sequence homology and variation among the fragments amplified from 3 RCC and 3 4*n*RR were analyzed using ClustalW software^1^.

### Fluorescence *in situ* Hybridization

The probes for fluorescence *in situ* hybridization (FISH) for the *5S* gene were constructed for RCC and amplified by PCR using the primers 5′-GCTATGCCCGATCTCGTCTGA-3′ and 5′-CAGGTTGGTATGGCCGTAAGC-3′. The FISH probes were produced by Dig-11-dUTP labeling (using a Nick Translation Kit, Roche, Germany) of purified PCR products. Purified PCR products of *5S* rDNA labeled with Dig-11-dUTP (Roche, Germany) were used as probes, and hybridization was performed according to the method described by Yi et al. (2003) with minor modifications. Purified PCR products of *5S* rDNA labeled with Dig-11-dUTP (Roche, Germany) were used as probes, and hybridization was performed according to the method described by Yi et al. (2003) with minor modifications. After treatment with 30 μg/ml RNase A in 2 × SSC for 30 min at 37°C, the slides with chromosome metaphase spreads were denatured in 70% deionized formamide/2 × SSC for 2 min at 70°C, dehydrated in a 70, 90, and 100% ethanol series for 5 min each (1 × SSC is 0.15 M NaCl/0.015 M sodium citrate, pH 7.6), and then air-dried. 4 μl of the hybridization mixture (approximately 100 ng of labeled probes, 50% formamide, 10 mg dextran sulfate/ml and 2 × SSC) was denatured for 10 min in boiling water, applied to the air-dried slides carrying denatured metaphase chromosomes under a 22 × 22 mm coverslip, and sealed with rubber cement. The slides were then put in a moist chamber and allowed to incubate overnight at 37°C.

Following overnight incubation, the coverslips were removed and the slides were rinsed at 43°C in: 2 × SSC with 50% formamide, twice, 15 min each; 2 × SSC, 5 min; 1 × SSC, 5 min, then air-dried. The spectrum signals were achieved by application of 8 μl of 5 μg/ml FITC-conjugated antidigoxigenin antibody from sheep (Roche, Germany) and a final incubation in the humidity chamber at 37°C. After a series of washes with TNT (containing 0.1 M Tris–HCl, 0.15 M NaCl, 0.05% Tween 20) at 43°C, the slides were mounted in antifade solution containing 2 μg/ml 4′, 6-diamidino-2-phenylindole (DAPI) for 5 min. Slides were viewed under a Leica inverted CW4000 microscope and a Leica LCS SP2 confocal image system (Leica, Germany). Metaphase spreads of chromosomes were analyzed in 10 RCC and 10 4*n*RR (20 metaphase spreads in each sample).

## Results

### Molecular Organization of the *5S* rDNA Classes

**FIGURE 1:**
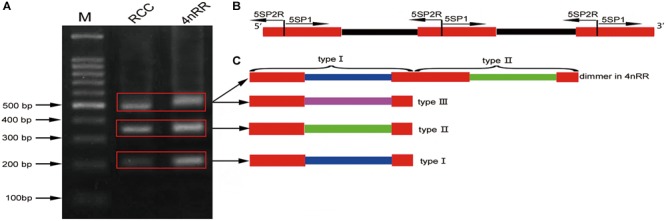
DNA bands amplified from RCC and 4*n*RR. **(A)** Using the primer pair *5S*P1-*5S*P2R, DNA fragments were amplified from RCC and 4*n*RR. M, DNA ladder markers (100 bp increments); lane 1, three DNA fragments (approximately 200, 340, and 500 bp) from RCC; lane 2, three DNA fragments (approximately 200, 340, and 500 bp) from the 4*n*RR; **(B)** Arrangement of higher eukaryotic *5S* rRNA genes (red) intercalated with non-transcribed DNA segments (NTS; black); **(C)** Representative sequences of *5S* rDNA type I, II, and III from RCC and 4*n*RR; red indicate *5S* rRNA genes; blue, green and purple indicate distinct NTS sequences.

**FIGURE 2:**
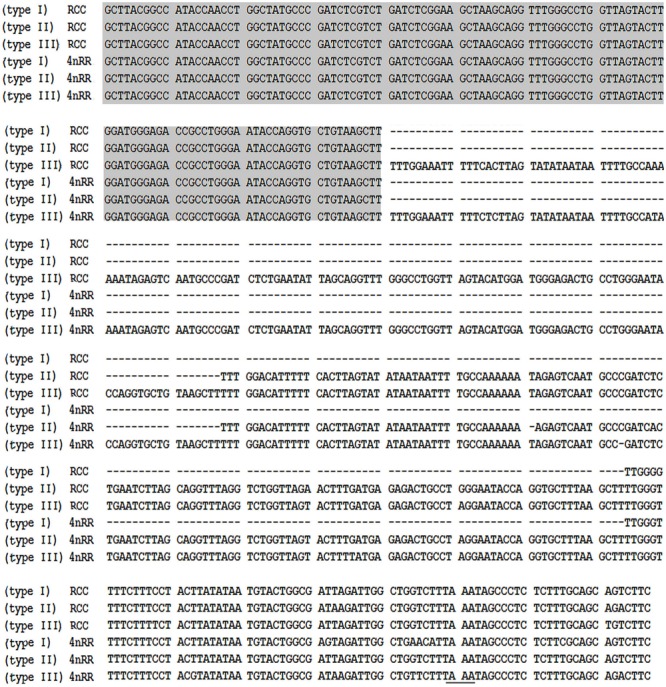
Representative sequences of *5S* rDNA from RCC and 4*n*RR. Complete *5S* coding regions are shaded; the NTS upstream TATA elements are underlined.

Using the primers *5S*P1 and *5S*P2R, fragments of approximately 200, 340, and 500 bp were generated from RCC and 4*n*RR (Figure 1A). All fragments proved to be *5S* rDNA sequences, each included the 3′ end of the coding region (pos. 1-21), the whole NTS region, and a large 5′ portion of the coding region of the adjacent unit (pos. 22-120; see Figure 1B). In RCC, the three types of *5S* rDNA classes (designated type I: 203 bp; type II: 340 bp; and type III: 477 bp) were characterized by distinct NTS types (designated NTS-I, NTS-II and NTS-III for the 83-, 220-, and 357-bp sequences, respectively; Figure 2). 4*n*RR had three types of *5S* rDNA classes, which were completely inherited from RCC (type I, type II and type III; Figure 2). All *5S* rDNA sequences have been submitted to GenBank, and their accession numbers are listed in Table 1.

**Table 1:** GenBank accession numbers of the *5S* rDNA sequences in RCC and 4*n*RR.

DNA fragments (bp)	GenBank accession numbers of the sequences	
	RCC	4*n*RR
203	GQ485555	MH44410
339, 340	GQ485556	MH44408
476, 477	GQ485557	MH44409

*RCC, Carassius auratus red var.; 4*n*RR, autotetraploid fish.*

**FIGURE 3:**
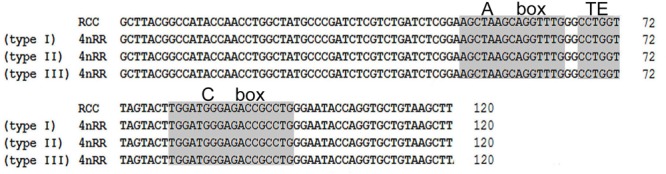
Comparison of *5S* coding regions from RCC and 4*n*RR. Internal control regions of the coding region are shaded.

**FIGURE 4:**
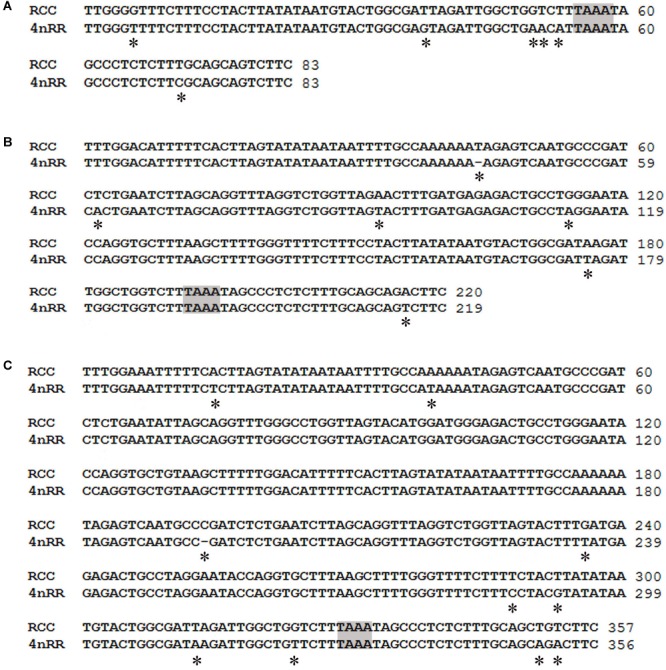
Comparison of the NTS sequences from RCC and 4*n*RR. **(A)** NTS-I sequences from RCC and 4*n*RR; **(B)** NTS-II from RCC and 4*n*RR; **(C)** NTS-III from RCC and 4*n*RR. The NTS upstream TATA elements are shaded; asterisks mark variable sites in the NTS.

Comparison of the 120-bp coding region of *5S* rDNA with those of RCC and 4*n*RR revealed great similarity (Figure 3). Nucleotide variation was not detected among the internal control regions (ICRs, i.e., the promoters for transcription) in 4*n*RR (Figure 3). A comparison of NTS-I revealed six base substitutions among the sequences (Figure 4A). A comparison of NTS-II showed five base substitutions and a deletion-insertion at position -177 (Figure 4B). A comparison of NTS-III elements showed nine base substitutions and a deletion-insertion at position -164 (Figure 4C). The above results indicate that obvious nucleotide variations were found in NTS sequences of 4*n*RR. In addition, characterization of the NTS-up stream region showed that the TATA control element, the regulatory region for *5S* gene transcription, was identifiable in the NTS of RCC and 4*n*RR (at -29 in all NTS sequences, where it was modified to TAAA; Figure 4), suggesting that all sequences analyzed here were likely to correspond to functional genes.

### Array Recombination of the *5S* rDNA Repeat Units

**FIGURE 5:**
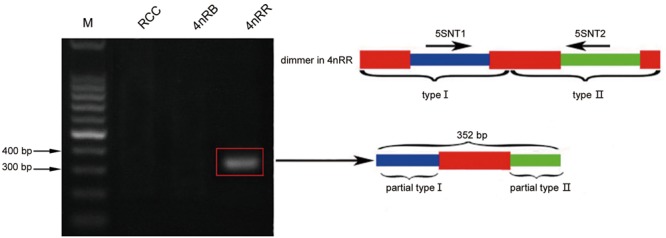
DNA bands amplified from 4*n*RR. Using the primer pair *5S*NT1-*5S*NT2R, DNA fragments were only amplified from 4*n*RR (approximately 352 bp); M, DNA ladder markers (100 bp increments); red indicates the *5S* rRNA gene, blue indicates NTS of type I, and green indicates NTS of type II.

Thirty clones of the 500 bp fragment from 4*n*RR were analyzed, and the sequence analysis revealed that five clones were dimeric *5S* rDNA formed by *5S* rDNA type I (the 99 bp gene sequence, 83 bp of NTS, and 21 bp gene sequence) and *5S* rDNA type II (the 99 bp gene sequence, 220 bp of NTS, and 21 bp gene sequence) (Figure 1C and Supplementary Figure 1). To verify whether the different *5S* rDNA classes (type I and type II) were associated within the same tandem array, we designed the primers *5S*NT1-*5S*NT2R. Using these primers, the PCR yielded a single band of 352 bp in 4*n*RR, but no band in RCC and 4*n*RB (Figure 5). Sequence analysis revealed that this fragment was formed by 72 bp of the type I (a 51 bp of the NTS and 21 bp gene sequence) and 280 bp of type II (the 99 bp of gene sequence and 181 bp of NTS) (Figure 5 and Supplementary Figure 2). The PCR amplification products of the two primers provided direct evidence to prove that the type I and type II repeats were associated within the same tandem array in 4*n*RR, suggesting that recombination of chromosomes occurred in the autotetraploid genome.

### Chromosomal Loci of *5S* rDNA

**FIGURE 6:**
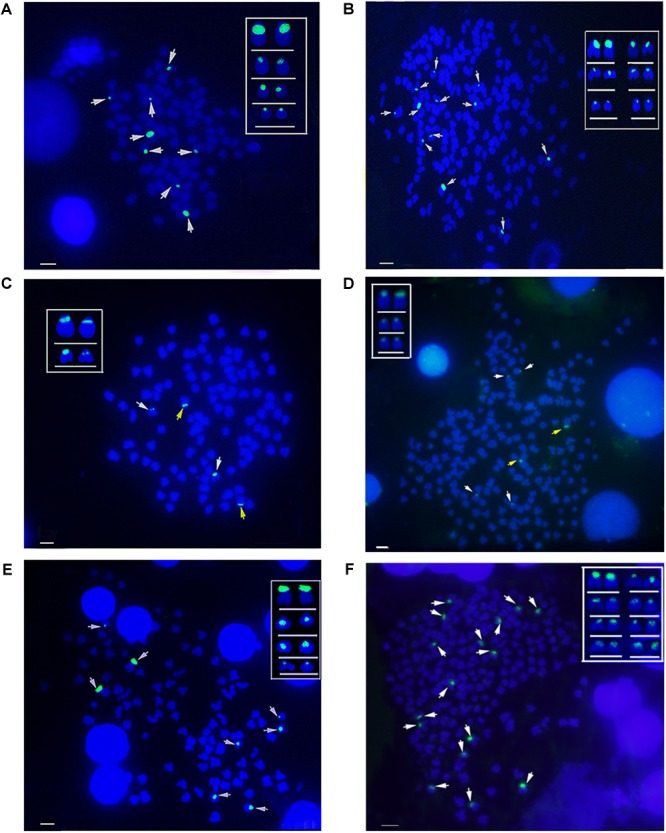
Examination of hybridizing signals by FISH in RCC and 4*n*RR. **(A)** Type I as probe showed eight *5S* gene loci (white arrows) in RCC; **(B)** Type I as probe showed twelve *5S* gene loci of type I (white arrows) in 4*n*RR; **(C)** Type II as probe showed two big (yellow arrows) and two small *5S* gene loci (white arrows) in RCC; **(D)** Type II as probe showed two big (yellow arrows) and four small *5S* gene loci (white arrows) in 4*n*RR; **(E)** Type III as probe showed eight *5S* gene loci (white arrows) in RCC; **(F)** Type III as probe sixteen *5S* gene loci (white arrows) in 4*n*RR. Bar = 3 μm.

The hybridization of type I *5S* rDNA probes showed eight *5S* gene loci in RCC chromosomal metaphases (Figure 6A and Table 2). Sixteen *5S* gene loci were expected in 4*n*RR chromosomal metaphases, but only twelve *5S* gene loci were found (Figure 6B and Table 2). Using type II *5S* rDNA as a probe, a pair of large *5S* gene loci was identified on homologous submetacentric chromosomes in RCC chromosomal metaphases, and a pair of small *5S* gene loci was localized on homologous subtelocentric chromosomes (Figure 6C and Table 2). In 4*n*RR chromosomal metaphases, a pair of large *5S* gene loci on a homologous submetacentric chromosome were found and other a pair of large *5S* gene loci on a homologous submetacentric chromosome were lost; two pairs of small *5S* gene loci was localized on homologous subtelocentric chromosomes (Figure 6D and Table 2). FISH hybridization of the type III *5S* rDNA probe to the RCC metaphase chromosomes yielded eight *5S* gene loci (Figure 6E and Table 2). As expected, sixteen *5S* gene loci were found in 4*n*RR chromosomal metaphases (Figure 6F and Table 2). The above results indicate that obvious loss of chromosomal loci occurred in 4*n*RR.

**Table 2:** Examination of chromosome locus numbers in RCC and 4*n*RR.

Fish type	No. of fish	No. of metaphase	Type I	Type II		Type III
			No. of loci	No. of big loci	No. of small loci	No. of loci
RCC	10	200	8	2	2	8
4*n*RR	10	200	12	2	4	16

*RCC, Carassius auratus red var.; 4*n*RR, autotetraploid fish.*

## Discussion

The evolution of *5S* rDNA is driven by birth-and-death processes with strongly purifying selection (Nei and Rooney, 2005; Pinhal et al., 2011; Vizoso et al., 2011), which can lead to the existence of different types of NTS (Pinhal et al., 2011). In teleosts, two distinct *5S* rDNA classes are characterized by distinct NTS types and base substitutions in the *5S* rRNA gene (Pendas et al., 1994; Moran et al., 1996; Martins et al., 2000; Wasko et al., 2001; Pinhal et al., 2009). Thus, possession of two *5S* rDNA classes seems to be a general trend for the organization of these sequences in the genomes of fish (Martins and Galetti, 2001). As ancient polyploidy fish, RCC have undergone an additional round of whole-genome duplication (Qin et al., 2016b). The origin of genic variants has been attributed to events of genome duplication followed by processes that result in the divergence of the duplicated sequences. Thus, RCC possess three distinct *5S* rDNA classes that are characterized by distinct types of NTS (Qin et al., 2010). In the current study, 4*n*RR derived from the distant hybridization of *C. auratus* red var. (2*n* = 100, RR) (♀) × *M. amblycephala* (2*n* = 48, BB) (♂), possess four sets of RCC-derived chromosomes and exhibit stability in chromosome number (or ploidy) over consecutive generations (F_1_–F_10_) (Qin et al., 2014b). 4*n*RR have three distinct *5S* rDNA classes that are completely inherited from RCC, but no new types of *5S* rDNA class were found, suggesting that divergence of the duplicated *5S* rDNA sequences were not fully formed in the early generations of the autotetraploid fish.

Because of incompatibility between parental chromosomes, allopolyploidization can increase genomic changes (Pontes et al., 2004). Our previous study revealed the influence of allopolyploidy on *5S* rDNA in fish, including parental genome specific loss, substitutions, and insertions-deletions in the NTS sequence (Qin et al., 2010, 2016a). Theoretically, homologous chromosomes should have high compatibility in autotetraploids. In this paper, however, obvious base variation and insertions-deletions of NTS were also observed in 4*n*RR, suggesting that autotetraploidization could lead to genetic variation in newly established autotetraploid genomes. Although there was genetic variation in NTS of *5S* rDNA, all sequences analyzed here were likely to correspond to functional genes, because they exhibited all the necessary features for correct gene expression: three ICRs (box A, internal element, and box C), a TATA control element, and a T-rich tail.

Autopolyploids are traditionally used to demonstrate multivalent pairing multivalent pairing during meiosis. However, the coexistence of four homologous chromosome sets does not result in multivalent formation during meiosis in 4*n*RR, and diploid-like chromosome pairing was restored (Qin et al., 2019). The presence of two distinct *5S* rDNA sequence types organized in different chromosomal regions or even on different chromosomes has been described for several fish (Pendas et al., 1994; Moran et al., 1996; Sajdak et al., 1998; Martins et al., 2002; Rodrigues et al., 2012; Qin et al., 2015b). In the current study, the different *5S* rDNA classes (type I and type II) were associated within the same tandem array in 4*n*RR. In addition, type I and type II *5S* rDNA clusters were localized in the chromosomes of 4*n*RR, and showed obvious loss of chromosomal loci. These findings are clear evidence that elimination of repetitive sequences and recombination of chromosomes occurred in newly established autotetraploid genomes. A positive linear relationship was found between increased bivalent pairing and elimination of specific, low-copy DNA sequences (Wendel, 2000). Thus, we speculate that the elimination of DNA sequences or recombination of chromosomes might generate immediate divergence between homologous chromosomes, providing a physical basis for diploid-like chromosome pairing in 4*n*RR.

## Ethics Statement

Fish treatments were carried out according to the regulations for protected wildlife and the Administration of Affairs Concerning Animal Experimentation, and approved by the Science and Technology Bureau of China. Approval from the Department of Wildlife Administration was not required for the experiments conducted in this manuscript. The fish were deeply anesthetized with 100 mg/L MS-222 (Sigma-Aldrich, St. Louis, MO, United States) before dissection.

## Author Contributions

QQ and SL designed the experiments. QL, CW, LC, YZ, HQ, and CZ performed the experiments. QQ and QL performed the statistical analysis. QQ wrote the manuscript. All authors read and approved the final manuscript.

## Conflict of Interest Statement

The authors declare that the research was conducted in the absence of any commercial or financial relationships that could be construed as a potential conflict of interest.

## Footnotes

1 http://www.ebi.ac.uk/clustalw/intex.html

## Abbreviations

4*n*RB: allotetraploid hybrids
4*n*RR: autotetraploid fish
BSB: blunt snout bream
FISH: fluorescence *in situ* hybridization
ICRs: internal control regions
NTS: non-transcribed spacer
PCR: polymerase chain reaction
RCC: red crucian carp
