# Supplementary material for: Molecular Organization and Chromosomal Localization Analysis of 5S rDNA Clusters in Autotetraploids Derived From Carassius auratus Red Var. (♀) × Megalobrama amblycephala (♂)
Source: Front Genet. 2019 May 15;10:437. doi: 10.3389/fgene.2019.00437 (PMC6529582; doi:10.3389/fgene.2019.00437)
Supplement: Supplementary file 1 [file Table_1.DOCX]

Supplementary Material

**Molecular organization and chromosomal localization analysis of *5S* rDNA clusters in autotetraploids derived from *Carassius auratus* red var. (♀) × *Megalobrama amblycephala* (♂)**

QinBo Qin, QiWen Liu, Chongqing Wang, Liu Cao, Yuwei Zhou, Huan Qin, Chun Zhao, ShaoJun Liu*

State Key Laboratory of Developmental Biology of Freshwater Fish, College of Life Sciences, Hunan Normal University, Changsha, 410081, Hunan, P.R. China.

^*^ Corresponding author:

Professor Shaojun Liu

[lsj@hunnu.edu.cn](mailto:lsj@hunnu.edu.cn)

## Supplementary Figures

**
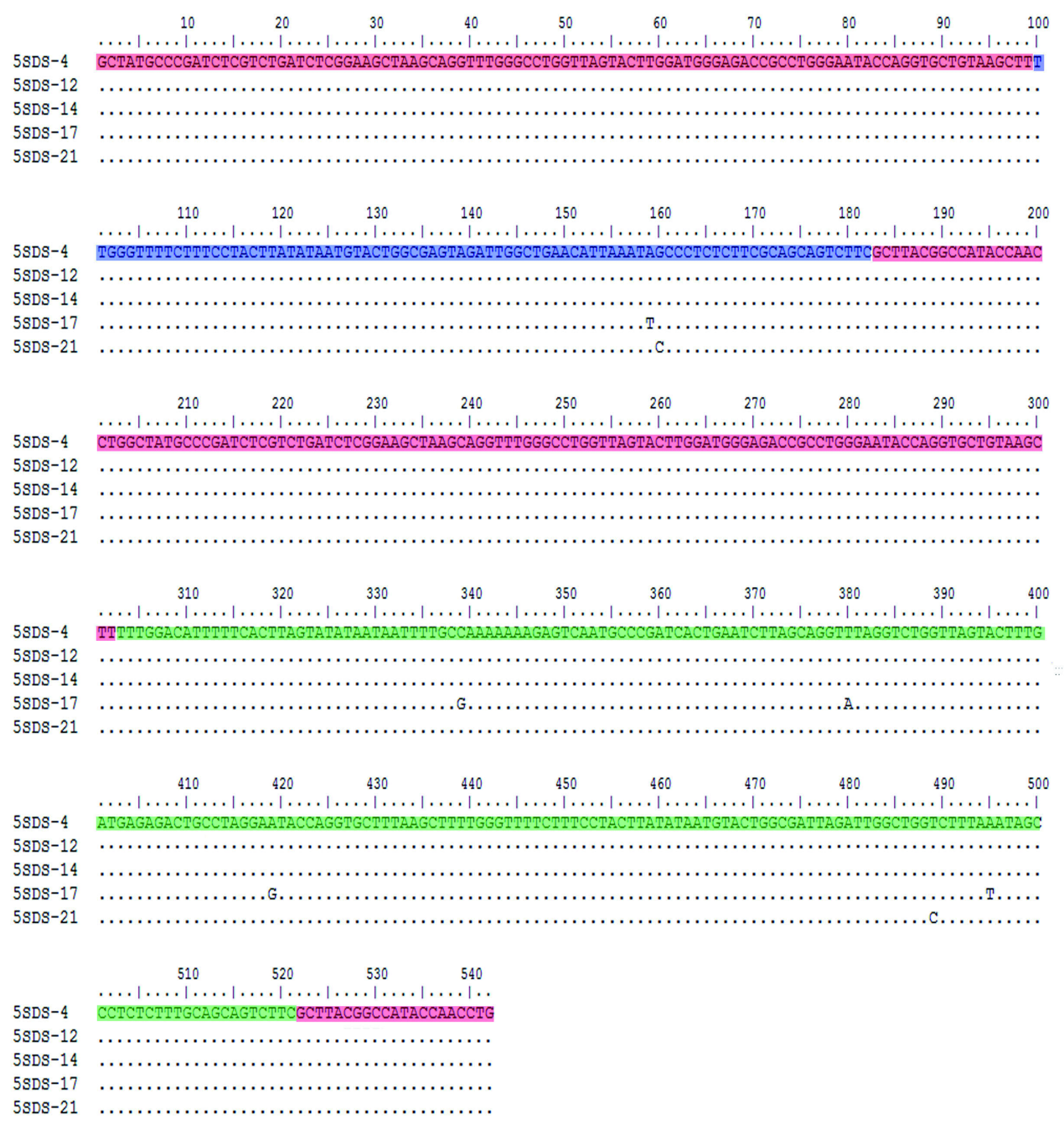
**

**Supplementary Figure 1. The type I and type II DNA sequences.**

Blue and green indicate NTS-I and NTS-II, respectively; red indicates *5S* rRNA gene sequence.


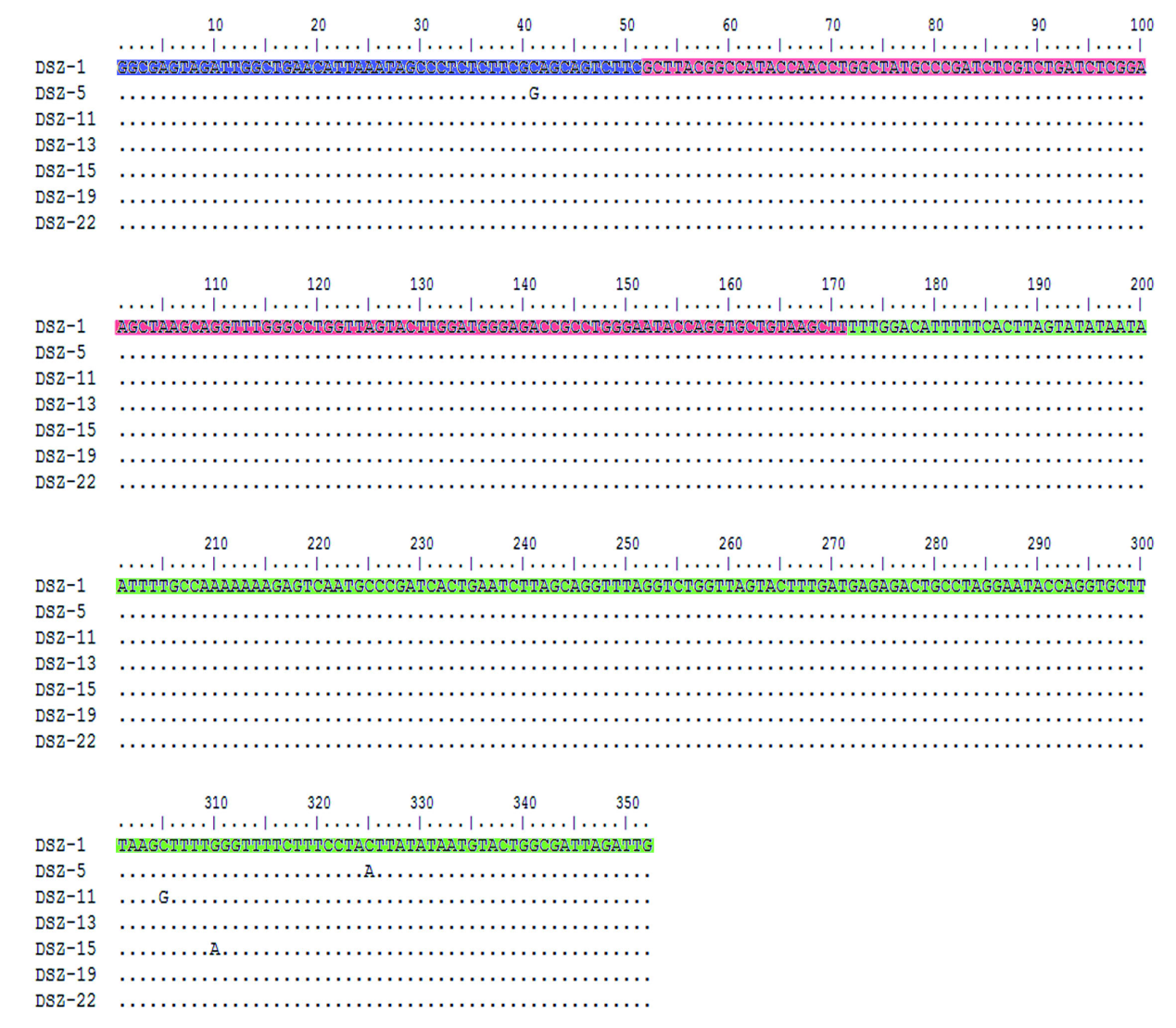


**Supplementary Figure 2.** **The 352 bp gene sequence amplified from 4nRR.**

Red indicates the 5S rRNA gene, blue indicates NTS of type I, and green indicates NTS of type II.
